# A review on exosomes application in clinical trials: perspective, questions, and challenges

**DOI:** 10.1186/s12964-022-00959-4

**Published:** 2022-09-19

**Authors:** Jafar Rezaie, Maryam Feghhi, Tahereh Etemadi

**Affiliations:** 1 Solid Tumor Research Center, Cellular and Molecular Medicine Institute, Urmia University of Medical Sciences, Shafa St, Ershad Blvd., P.O. BoX: 1138, Urmia, 57147 Iran; 2 Institute of Molecular Biophysics, Florida State University, Florida, USA; 3 Department of Biology, Faculty of Science, Arak University, Arak, Iran

**Keywords:** Exosomes, Extracellular vesicles, Clinical trials, Exosome therapy

## Abstract

**Background:**

Exosomes are progressively known as significant mediators of cell-to-cell communication. They convey active biomolecules to target cells and have vital functions in several physiological and pathological processes, and show substantial promise as novel treatment strategies for diseases.

**Methods:**

In this review study, we studied numerous articles over the past two decades published on application of exosomes in different diseases as well as on perspective and challenges in this field.

**Results:**

The main clinical application of exosomes are using them as a biomarker, cell-free therapeutic agents, drug delivery carriers, basic analysis for exosome kinetics, and cancer vaccine. Different exosomes from human or plant sources are utilized in various clinical trials. Most researchers used exosomes from the circulatory system for biomarker experiments. Mesenchymal stem cells (MSCs) and dendritic cells (DCs) are two widely held cell sources for exosome use. MSCs-derived exosomes are commonly used for inflammation treatment and drug delivery, while DCs-exosomes are used to induce inflammation response in cancer patients. However, the clinical application of exosomes faces various questions and challenges. In addition, translation of exosome-based clinical trials is required to conform to specific good manufacturing practices (GMP). In this review, we summarize exosomes in the clinical trials according to the type of application and disease. We also address the main questions and challenges regarding exosome kinetics and clinical applications.

**Conclusions:**

Exosomes are promising platforms for treatment of many diseases in clinical trials. This exciting field is developing hastily, understanding of the underlying mechanisms that direct the various observed roles of exosomes remains far from complete and needs further multidisciplinary research in working with these small vesicles.

**Video Abstract**

**Supplementary Information:**

The online version contains supplementary material available at 10.1186/s12964-022-00959-4.

## Background

**Fig. 1:**
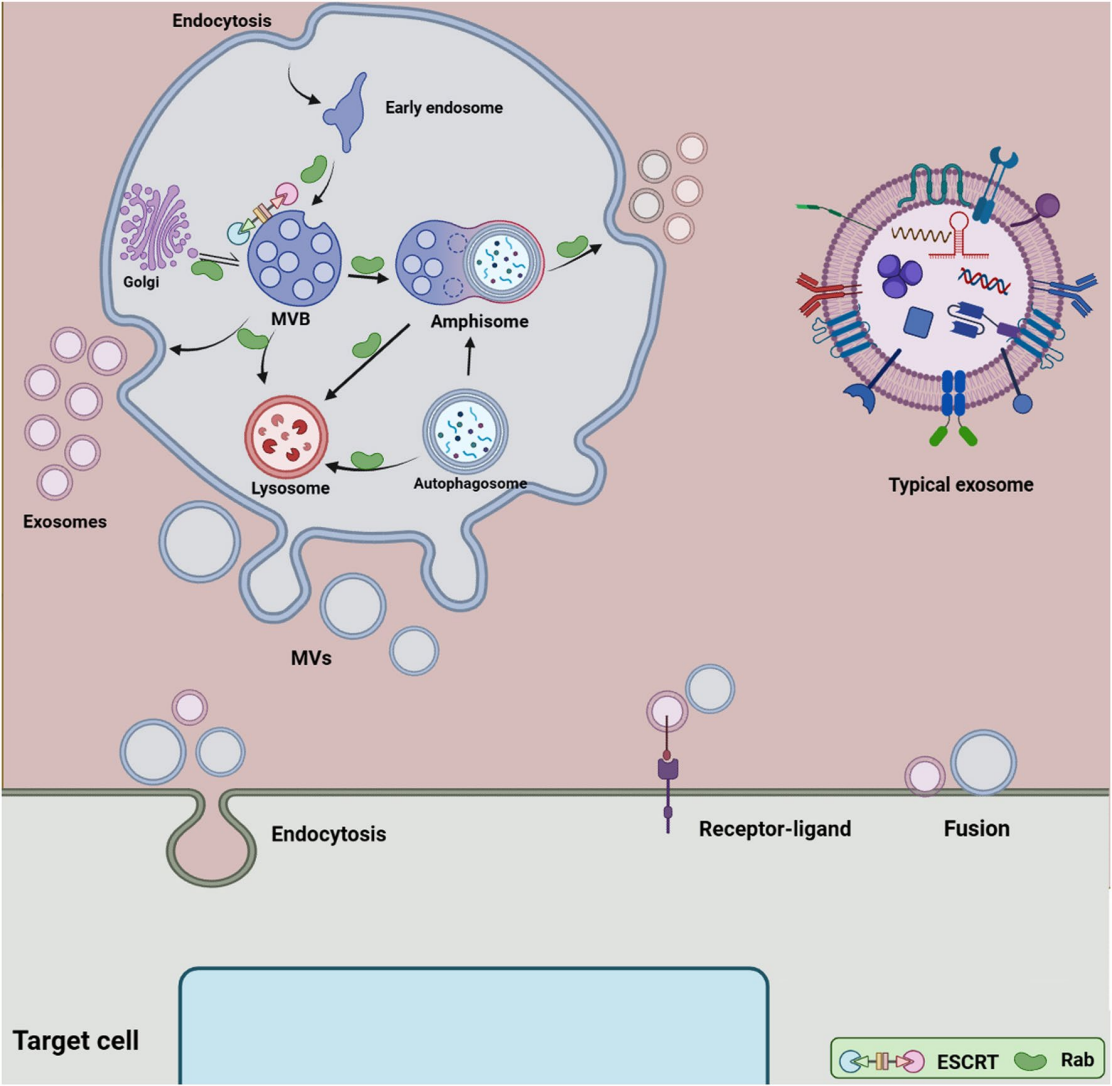
Extracellular vesicles (EVs) biogenesis. Two main subclasses of EVs are exosomes and microvesicles (MVs). Exosomes biogenesis is complex and occurs inside multivesicular bodies (MVBs) located in the cytoplasm. ESCRT complexes and different molecules contribute to loading, sorting and forming exosomes in ATP dependent and independent manners. Exosomes cargo come from Golgi apparatus, the endosomal pathway, and the cytoplasm. Rab proteins mediate intracellular trafficking of endosomal compartments and exosomes. MVBs have been reported to fuse with either plasma membrane, lysosomes or amphisoms. When MVBs fuse with the plasma membrane, exosomes are released into the extracellular milieu. There is evidence that exosomes biogenesis pathway may crosstalk with autophagy flux. MVBs may fuse with autophagosomes and form the hybrid vesicles known ‘’Amphisomes’’, which finally fuse with the plasma membrane or lysosomes. MVs are larger than exosomes and formed from the plasma membrane through the outward budding and shedding process. EVs can affect the target cells' fate and signaling pathways in three possible ways comprising endocytosis, receptor-ligand interaction, and direct fusion with the plasma membrane

Extracellular vesicles (EVs) include heterogeneous cell-derived vesicles produced by most cells via diverse mechanisms that contribute to cell-to-cell communication by transferring biological signals such as many non-coding RNAs, coding RNAs, DNA strands, portions, and lipids to counterpart cells [1]. EVs can also deliver their cargo/ messages to target cells by many body fluids including, the circulating system, saliva, cerebrospinal fluid (CSF), urine, milk, and bronchoalveolar lavage fluid (BALF) [2]. Once delivered to target cells, EVs can regulate the fate and morphology of recipient cells by driving different signaling pathways, which may result in either normal physiology or pathogenesis depending on the origin of EVs and the state of cells. Emerging evidence suggests that EVs play pivotal roles in a range of cellular processes including, proliferation, growth, development, angiogenesis, immunomodulation, infection, metastasis, reprogramming and remodeling [3–6]. Three major subclasses of EVs have been documented including microvesicles (MVs) or ectosomes, apoptotic bodies, and exosomes [7, 8] (Fig. 1) (Table 1). The term MVs refers to EVs that are generated from the plasma membrane with a size range 100–1000 nm via a complex mechanism known as shedding or blebeling of the plasma membrane resembling a virus outward budding a cellular membrane [7, 8]. Another EVs that have increasingly attracted scientists' attention is exosomes with a size range 30–150 nm [7, 8]. These vesicles have been reported are also heterogeneous both in size and cargo consequently functioning diversely on target cell signaling [9]. The origin of exosomes is vesicles involved in the endocytosis pathway were a type of late endosomes named multivesicular bodies (MVBs) begin to generate exosomes. Recent progress in EVs has shown that exosomes biogenesis may interplay with another endomembrane system to maintain cellular homeostasis [10]. For example, crosstalk between exosomes signaling pathway and autophagy has been reported in normal physiology and several diseases [11]. Over the past decade, there has been a dramatic increase in translational medicine of exosomes seeking to improve the outcome of therapies and focus on precision medicine [12, 13]. Exosomes represent unique properties that engrossed the researchers to use them for the treatment of many diseases including degenerative disorders and cancers [14, 15]. Until now, many clinical trials are carried out using exosomes from different sources for various diseases. Although this approach is interesting and clinical trials showed satisfactory outcomes, there are some challenges and limitations in this field and further investigations are needed to validate these results and introduce a gold standard for exosomes-based studies. Methodical examination of the efficiency and safety of exosomes needs determination of their individuality and purity. The International Society for Extracellular Vesicles (ISEV) instigated the standardization of EVs isolation and characterization procedures by releasing MISEV guidelines in 2014 and 2018, which discuss the most essential characteristics that must be considered in EVs-based studies [16, 17]. Furthermore, the EV-TRACK database was established in 2017 that offers researchers to credit their purification and characterization methods before publication and make recommendations on possible limitations of the experimental plan (https://evtrack.org/about.php). This paper is an overview of the potential clinical application of exosomes and the challenges and questions in this roadmap.

**Table 1:** Types of extracellular vesicles

EVs	Size	Markers	Mechanism of biogenesis
Exosomes	30–150 nm	CD63, CD9, CD81, Tsg101	Generated by inward budding of the membrane of MVBs through ESCRT-dependent Or/and ESCRT-independent and released into the ECM upon fusion of MVBs with the plasma membrane
Exomeres	< 50 nm	Unknown	Unknown
Microvesicles or ectosomes	100–1000 nm	ARF6, Annexin A1	pinching off from membrane protrusions/the plasma membrane shedding
Apoptotic bodies	50–5000 nm	Phosphatidylserine	Generated from apoptotic cells following stimulation of apoptosis-related pathways

## Exosomes

**Fig. 2:**
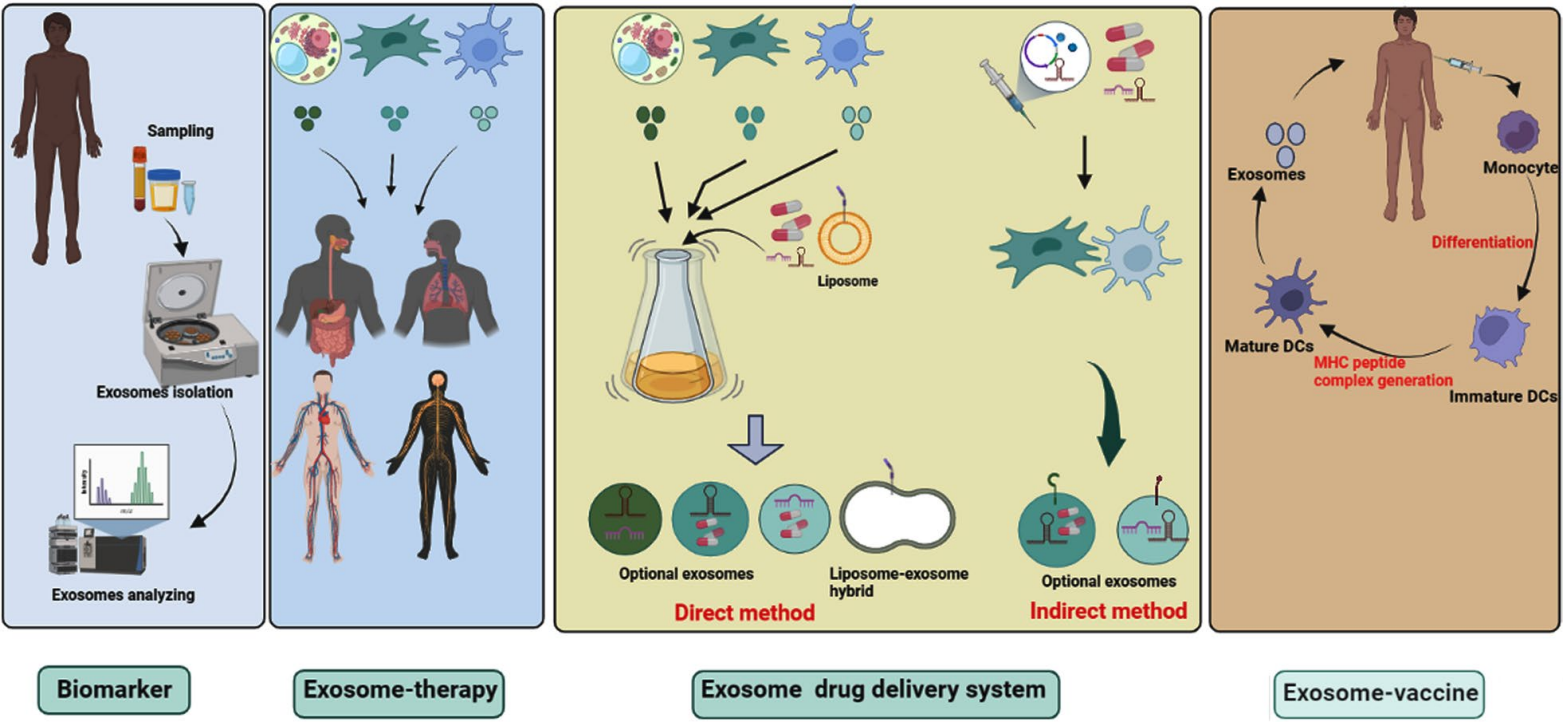
Clinical application of exosomes. In clinical trials, exosomes are being used as biomarkers, cell-free therapy (exosome-therapy), drug delivery system, and cancer vaccine. Exosomes from plant cells, mesenchymal cells, T cells, and dendritic cells are used for the treatment of different diseases. In addition, exosomes from these sources are promising carriers for drug delivery systems. In the direct method, exosomes are loaded with therapeutic agents, while through indirect methods, proper cells are genetically engineered or co-cultured with therapeutic agents to produce artificial exosomes

The term exosomes refer to membrane-bound vesicles released from many cells into the extracellular matrix [7, 18]. The main idea is that these nano-particles are generated inside the cells, within the MVBs, by the invagination of the membrane of MVBs, which leads to the generation of intraluminal vesicles (ILVs) inside MVBs [7, 18] (Fig. 1). These vesicles may be named pre-exosomes, which can be secreted out of cells following the fusion of MVBs with the cellular membrane; now there are known as exosomes [7, 18]. Always MVBs do not expel ILVs out of cells, rather they may fuse with the lysosomes and subsequently ILVs and their cargo are degraded for recycling and reuse for cellular homeostasis [7, 18]. In addition, there is evidence that MVBs may fuse with the autophagosomes of the autophagy pathway, and generate hybrid vesicles known as ‘’Amphisomes’’, subsequently, amphisomes may now fuse with the plasma membrane and secrete exosomes with other cargo [19, 20]. Understanding how and why MVBs fuse with different membranes has been a long-standing goal because it is critical to discriminate heterogeneity in exosomes, and relevant physiological consequences, regulate their production for clinic use and monitor pathological conditions. Various molecules and complexes are present on MVBs membrane that mediate cargo loading and sorting inward budding membrane, and pinch off newly formed ILVs into MVBs [7, 18]. For instance, an ESCRT-related mechanism, consists of four complexes, that mediate biogenesis and loading ILVs using ATP molecules in a regulated manner or an ESCRT-independent mechanism is involved [7, 18]. Many Rab proteins linked with MVBs facilitate the movement of MVBs in different ways [7, 18]. Among them, the role of Rab7, Rab8, Rab11, Rab27, and Rab35 in regulating the exosomes pathway has been recognized [21–24]. The SNARE proteins are short molecules that drive the fusion of MVBs and the cellular plasma [21, 22]. Upon secretion into the cellular space, exosomes interact with the target cells those either nearly located or distantly. It was suggested that three mechanisms that exosome or other EVs recruit to affect recipient cells' function comprising, endocytosis (e.g. phagocytosis and pinocytosis), receptor-ligand interaction, and direct-fusion, which cause changes in cellular and biological processes, participating in normal physiology or worsening pathological condition [6, 25]. This is an exosomes journey; however, further investigations would be needed to determine exactly how exosomes are generated for a definite purpose. Exosomes cargo comprises molecules provided from the cellular membrane, endosomal compartments, the cytoplasm, and those that come from the endomembrane system like the Golgi apparatus [7, 18]. Exosomes exhibit a specific biconcave or cup-like shape during the drying process, while they appear spheroid under transmission electron microscopy [26]. These vesicles have a density 1.08–1.19 g/ml with common exosomal markers such as CD9, CD63, CD82, CD81, Alix, and Tsg101 [9, 27]. Thousands of types of biomolecule are present within exosomes, comprising proteins, numerous RNAs, and lipids whose data are collected and presented by various databases such as Vesiclepedia (http://www.microvesicles.org), Exocarta (http://www.exocarta.org), and a Bioinformatics lab from china (http://bioinfo.life.hust.edu.cn). Understanding mechanisms that drive exosomes biogenesis and loading, as well as exosomes cargo and ways of exosomes uptake by other cells, will support scientists to achieve meticulous and efficient translational medicine. It is worthy to note that full comprehension of the nature, purity, and origin of exosomes would be critical for downstream experiments. For example, exosomes from stem cells may participate in regeneration and normal physiology, while those derived from infected or cancer cells mediate pathogenesis [28, 29] (Fig. 2).

**Box 1:** Key questions about exosomes biogenesis

Are underlying mechanisms in vitro relevant in vivo for exosomes biogenesis? Do different cells use the same mechanisms for exosomes biogenesis? What is the main regulation mechanism for the sorting specific exosomal cargo? Do environmental signals such as extracellular matrix and receptor-ligand interaction affect exosomes biogenesis? What is the causative reason that MVBs choose to fuse with lysosomes? Do exist a subpopulation of MVBs inside typical cells? How crosstalk between exosomes and other signaling pathways is regulated? For example, autophagy, apoptosis, and traditional secretory pathways may interplay and share function with exosomes, therefore the exact adjustment mechanism for exosomes biogenesis remain an obstacle in stress condition What is the main way that exosomes is used to affect target cells? In another word, do all cells use a common way to affect target cells?	

## Clinical trials

**Fig. 3:**
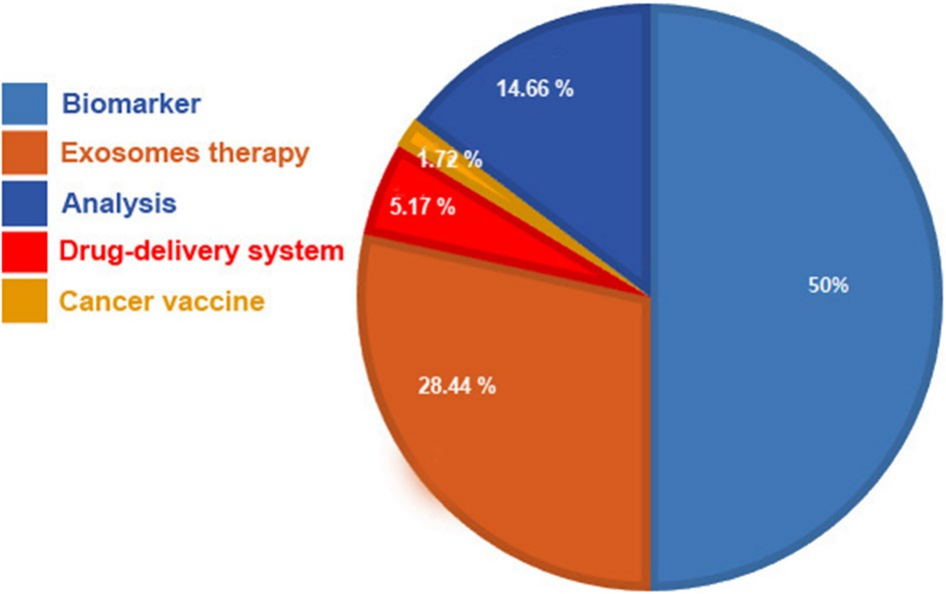
Analysis of exosome-based clinical trials

**Fig. 4:**
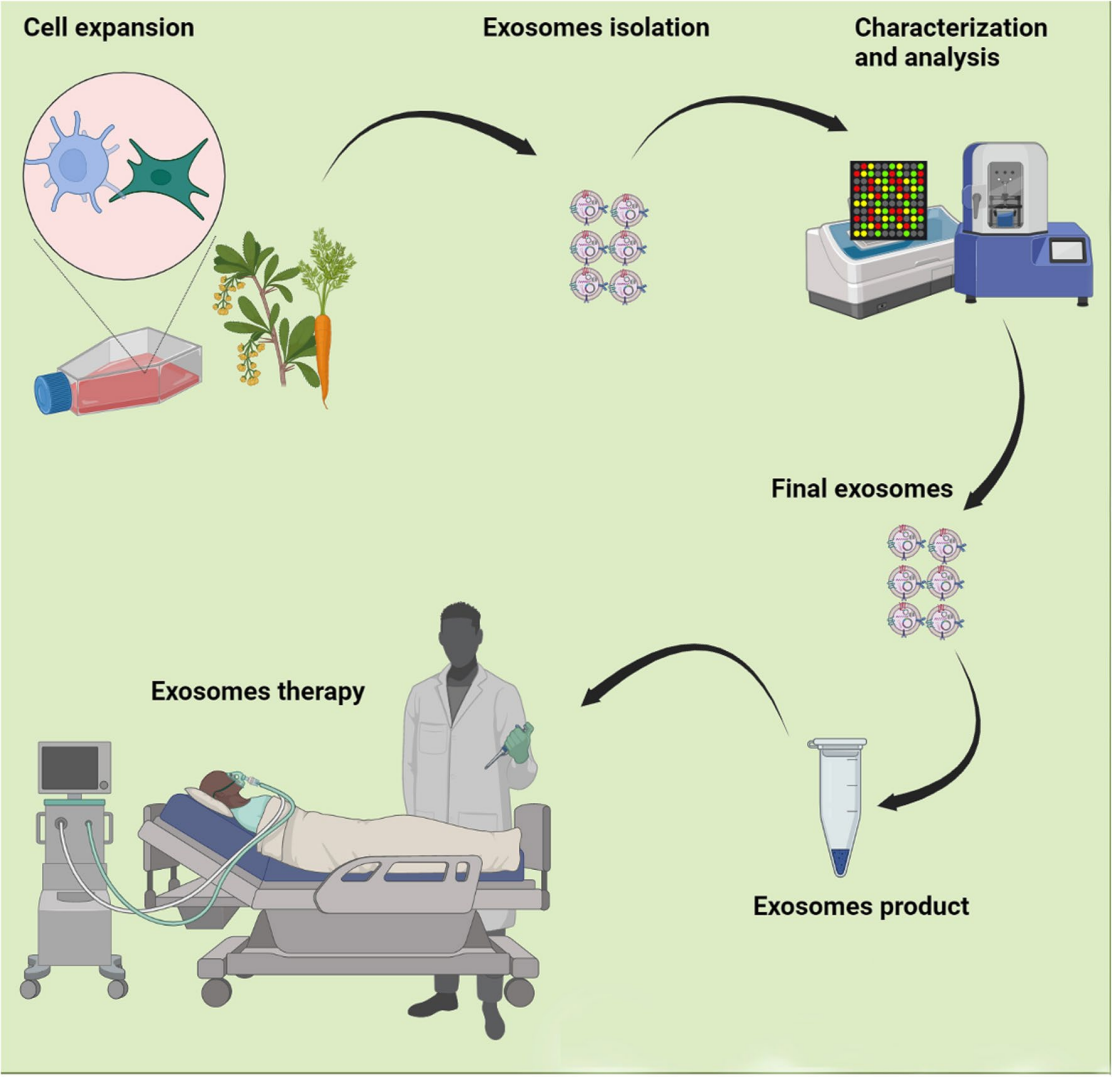
Flowchart for current Good Manufacturing Practices (GMP) manufacturing of exosome therapeutic products

It is well-established that exosomes are an ideal candidate for the treatment of many diseases. Before clinical trials, many preclinical experiments have confirmed the advantages of exosomes for the treatment of many diseases spanning the field of regenerative medicine to cancers [28, 29]. Over the recent years, exosomes were investigated in clinical trials, collectively two types of exosomes are used in clinical trials, specifically, exosomes of human cells/samples and plants specimen. A survey on ClinicalTrials.gov (https://clinicaltrials.gov/) shows the major applications of exosomes are biomarkers, exosome-therapy, drug delivery systems, and cancer vaccines (Fig. 3). Besides, some trials have been aimed to analyze exosomes from human samples under different conditions. An analysis shows that a total of 116 trials have been recorded, of which 58 (50%) belong to biomarker applications (Fig. 3). In the case of exosome therapy, 33 (28.44%) studies have been registered (Fig. 4). For drug-delivery system trials 6 (5.17%) studies, while for exosomes basic analysis 17 (14.66%) studies have been registered (Fig. 4). Finally, 2 clinical trials (1.72%) are related to exosome vaccine studies. Exosomes in clinical trials need to comply with good manufacturing practice (GMP). A GMP-grade exosome production method comprises the type of cells, culture environment, cultivation system, and culture medium. Further purification is essential after production, usually divided into three-step process. The third subject in GMP of exosomes is the establishing of characterization and identification method, comprising physical configuration and bioactivity function characteristics [30]. Furthermore, European Medicines Agency (https://www.ema.europa.eu/en) has released scientific recommendations on classification of advanced therapy medicinal products. This agency describes that new scientific progress in cellular and molecular biotechnology has led to the development of advanced therapies, such as gene therapy, somatic cell therapy, and tissue engineering. This nascent field of biomedicine offers new opportunities for the treatment of diseases and dysfunctions of the human body. In addition, it provides guidelines, recommendations, and criteria for researchers to advanced therapy medicinal products and amending directive and regulation [31, 32].

## Biomarker application

As mentioned above, exosomes are present in many biofluids, possibly providing a new window into monitoring the status of cells in normal physiology or upon onset of pathological conditions [33, 34]. By a simple liquid biopsy of plasma, serum, urine, saliva etc. exosomes and other EVs are available for profiling their cargo for diagnosis, prognosis, progress, and chemoresistance biomarker applications [33, 34]. Even though most cell types release exosomes, evidence supports the up-regulated exosome secretion in several pathological conditions like cancer [35]. Exosomes are stable in most body fluids and shield biomolecules from degradation. Under pathological conditions, cellular changes can be reflected in the biological component of exosomes released by cells. Altered cargo of exosomes can be identified and analyzed by transcriptomics, proteomics, and lipidomics investigations; therefore, the discrepancy in levels of specific molecules would be useful for biomarker application [36]. Different laborites have shown differential expression of miRNAs in cancer cells and their exosomes in vitro, in addition, some studies conducted on ex-vivo and animal cancer models have confirmed alternation in the expression level of miRNAs [37, 38]. Different miRNAs are being developed, including miRNA-200-5p, miRNA-378a, miRNA-139-5p, and miRNA-379 for lung carcinoma [39] and miRNA-21 for oesophageal squamous cell carcinoma [40] have been suggested as non-invasive diagnostic biomarkers. Besides, exosomal proteins have biomarker potential for many cancers [41]. In the case of cancers, the early detection of a tumor is vital for effective therapy [42]. As shown in Fig. 4A and B, among them 43 (74.13%) clinical trials related to cancer biomarker studies, the rest belongs to other diseases (about 25.87%). A well-understanding of exosomal cargo sorting mechanism and secretion, transferring them through specific body fluids, and their stable state levels during physiological settings and under different disease contexts would result in more strong biomarkers for monitoring disease onset and development [43, 44]. In addition, further studies are essential to validate the specificity and sensitivity of exosomal biomarkers against traditional cancer biomarkers.

**Box 2:** Key questions about exosomes-based biomarker

Do preclinical results relevant to clinical trial findings? Does cargo heterogeneity of exosomes cause bias in results? Because the exosome populations expressed from single cells are heterogeneous, the content concentrations are expected to exist in a range and not at a set standard What is/are a common/s biomarker for a range of diseases? What is the contribution of genetics to different outcomes? In other words, Do racial factors correlate with the type of exosomal cargo at the same pathological condition? Do exosomes contain biomolecules or properties that protect their cargo from degradation? How do such conditions affect the loading mechanism of exosomes?	

## Drug-delivery application

Exosomes as developed biological nanoplatforms for delivering therapeutic agents have been examined in preclinical and clinical experiments [45]. In the field of nanotechnology, designing smart carriers for targeted drug delivery is a gold standard [46]. Exosomes may be a good applicant for the clinical translation of several drug-delivery platform formulations [45]. From a drug delivery standpoint, exosomes are analogous to liposomes, regarding phospholipid structure. However, exosomes can be collected from various body fluids and cells with a complex structure of different lipids and surface proteins and receptors; these biomolecules may help tissue and cell targeting, whereas some warrant minimal non-targeting interactions [47, 48]. These distinctive protein-decorated phospholipid membranes may likely have the definite barcodes desirable to interact with their target both neighboring and at distant locations. Even though extensive investigations, the superiority of exosome-based drug delivery system over delivery by other synthetic nanocarriers, like liposomes, and the related benefit-risk ratio remain problems of debate [49]. Therapeutics agents, including nucleic acid, proteins, and drugs, can be inserted into exosomes at least in two general methods and then carried to a specific target [45, 50]. These methods are direct methods or exogenous approaches where drugs are loaded into isolated exosomes via several methods; and indirect methods or endogenous approaches in which parental cells are loaded with drugs or genetically modified for expressing optional proteins, receptors, and RNAs. The endogenous approaches has a lower level of complication if the parent cells right produce exosomes with the chosen molecule. Alternatively, there is a method known as exosomes-inspired liposomes in which a desired liposome is fused with exosome to construct a hybrid carrier [51]. Ongoing clinical trials show that exosomes are being used for delivering therapeutic agents, for example, there are six clinical trials registered in ClinicalTrials.gov resource (https://clinicaltrials.gov/ct2/home). In two clinical trial, authors aimed to encapsulate curcumin into plant derived exosomes for treatment colon cancer and Irritable Bowel Disease (NCT01294072 and NCT04879810) by direct methods. In addition, three clinical trials aimed to modify mesenchymal stem cells (MSCs) for producing overexpressing exosomes (Table 2). None of these trials is completed and results are not available until now. It is worthy to note that some clinical trials used microparticles from tumor cells for delivering drugs (e.g. NCT02657460 and NCT01854866). These studies used the term microparticles not exosomes and are recorded before the ISEV guidelines for EVs-based studies.

**Table 2:** Clinical trials related to exosomes-based drug delivery

Exosomes	Condition	Cargo	Phase	Recruitment Status	Number
Plant (Grape)	Normal and Colon Cancer Tissue	Curcumin	I	Recruiting	NCT01294072
Plant (Ginger)	Irritable Bowel Disease	Curcumin	Not Applicable	Recruiting	NCT04879810
T-REx™-293 cells	SARS-CoV-2 PNEUMONIA	CD24	I	Recruiting	NCT04747574
MSCs	Familial Hypercholesterolemia	Ldlr mRNA	I	Not yet recruiting	NCT05043181
MSCs	Pancreatic Adenocarcinoma	KRAS G12D siRNA	I	Recruiting	NCT03608631
MSCs	Cerebrovascular Disorders	miRNA-124	I	Recruiting	NCT03384433

Although researchers have endeavoured to harness the exclusive properties of exosomes to advance smart drug delivery systems that show considerable profits in pharmacokinetics, targeting, and safety against those of synthetic nanocarriers, clinical translation of these results faces challenging [52]. Because of the characteristic complexity of the exosomes themselves, size heterogeneity, and natural (batch-to-batch) discrepancies run throughout their assembly, the intrinsic risks of the biogenesis procedure are higher than those of virginally synthetic fabrication approaches [53, 54]. Furthermore, technologically and reproducible available approaches are required to load them with therapeutic drugs [53]. Even though loading approaches for liposomes are optimized and used in an industrial context, such methods for exosomes are still needed. Current cell culture and exosome purification methods limit the implementation of standardized and large-scale production of exosomes [55]. Therefore, for exosomes to be considered as a reliable therapeutic carriers, scalable manufacturing processes are required to produce exosomes in a fast, cost-effective, and reproducible way.

**Box 3:** Key questions about exosomes-based drug deliveryKey questions about exosomes-based biomarker

How do exosomes interact with various milieus and are sustained or removed in body fluids? What is/are standardized technique/s for the isolation and purification of exosomes? Emerging a fast and accurate method of exosomes isolation is one of the most vital tasks in the current field of research Which exosomes are appropriate for designing drug carriers? Exosomes from stem cells, although safe, may contribute to promoting tumor growth or tumor derived exosomes may induce tumorigenesis rather than therapy. In the case of cancer, using tumor-derived exosomes may represent more advantages than those of stem cells due to their targeted homing and immunactivation potential. In addition, exosomes isolated from cell cultures may vary and show unpredictable properties even when the same type donor cells were used. Current cell culture and exosome purification methods limit the application of standardized and mass production of exosomes Which method/s is/are applicable for the drug delivery system? Which method is gold-standard for inserting drugs into exosomes? Different mechanical or chemical techniques including electroporation, incubation, sonication, and saponin are being used to incorporate drugs into exosomes; selecting each method may depend on the study design, source of exosomes, and types of drug Do loading methods damage exosomes entity and functions? What is the non-targeting effect of engineered exosomes? Genetically modifying may affect the function and nature of exosome-producing cells, resulting in unwanted exosomes	

## Exosomes therapy

**Fig. 5:**
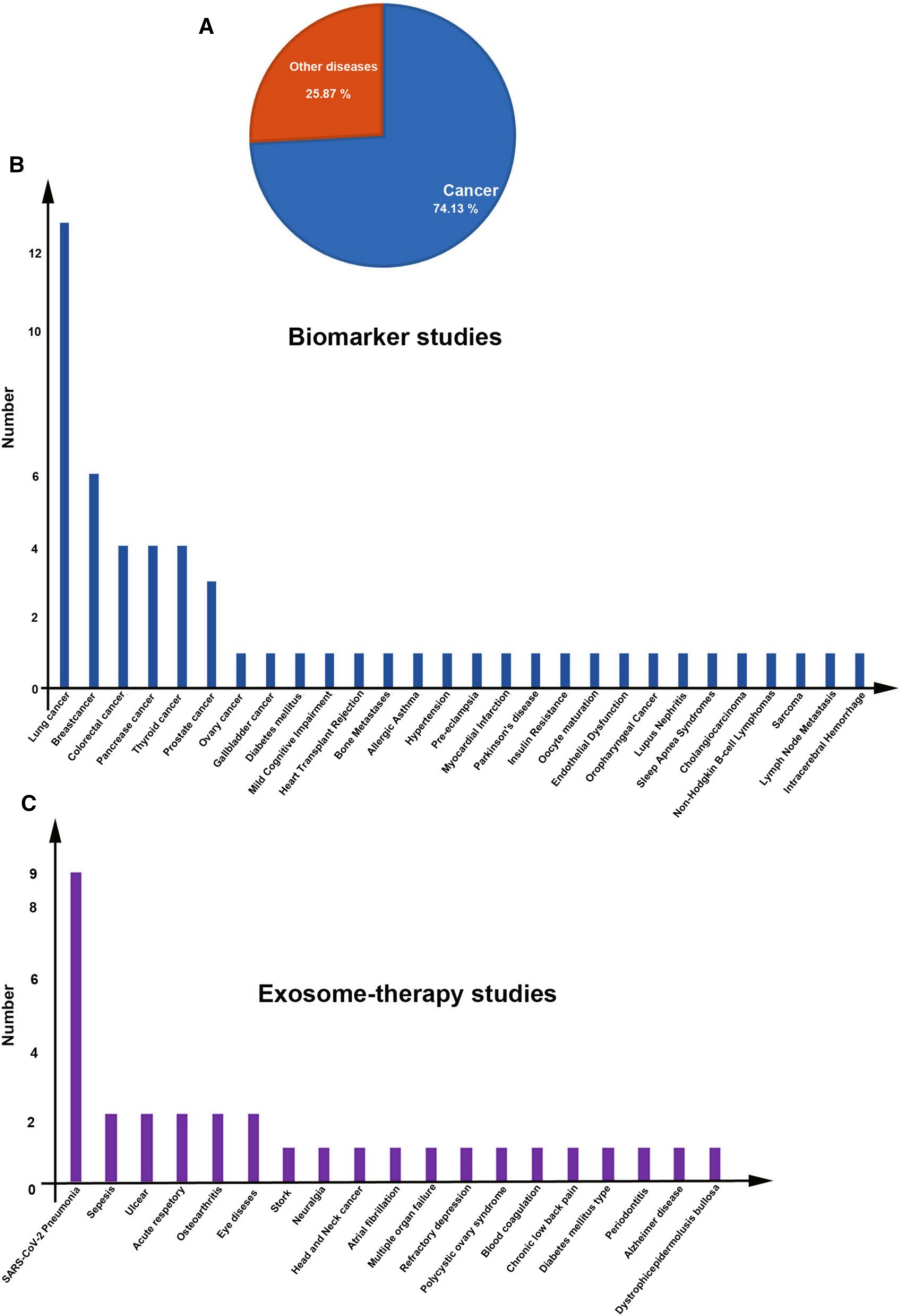
Analysis of exosome-based biomarker clinical trials (**A**, **B**) and exosome therapy-based clinical trials for different diseases (**C**)

Today, there is increasing interest in using cell derivatives such as exosomes rather than stem cells for therapies [56, 57]. Exosomes from stem cells represent the same therapeutic and clinical benefits as stem cells exert [58]. Currently, known exosomes from many native stem cells have been shown to recover adversarial conditions and damaged tissues. Some laboratories used strategies to increase exosomes production and improve the therapeutic potential of exosomes, including preconditioning of stem cells, genetically modification of stem cells, and conjunction of exosomes with biomaterials [59–61]. For example, norepinephrine and *N*-methyldopamine are used to promote exosomes secretion from MSCs without changing their modulatory ability [62]. Other physical strategies comprise incubation of stem cells with hypoxia, acidic medium, and lipopolysaccharides. In addition to animal cells, microbial and plant-derived exosomes have been to have [61]. However, the safety of these exosomes/EVs needs to be evaluated. In the clinical context, exosomes are registered for the treatment of several diseases. In recent years, because of outbreeding COVID-19 pneumonia; MSCs-derived exosomes are being increasingly examined owing to their immunomodulation and regenerative potential. According to ClinicalTrials.gov resource (https://clinicaltrials.gov/ct2/home), a total of 33 exosome-therapy clinical trials are registered of which 20 clinical trials used MSCs-derived exosomes (60.60%). Researchers also used Plant, T cells, and DCs derived exosomes for the treatment of different diseases. Most studies (9 clinical trials) belong to SARS-CoV-2 pneumonia treatment (Fig. 4). These results indicate that exosomes from MSCs are frequently being used for regenerative medicine. However, some studies indicated that MSCs exosome therapy is occasionally discussed as a “double-edged sword”[63, 64]. Contrary to satisfactory results, safety, side effects, and unwanted results of exosome therapy are not fully clear [65]. A clinical study aimed to investigate the ability of plant (grape) exosomes to prevent oral mucositis associated with chemoradiation treatment of head and neck cancer (NCT01668849). Recently, the FDA indicated that patients in Nebraska who were treated with unapproved products advertised as exosomes based products reported severe adversative effects. The agency highlighted that there are presently no governing approved exosomes products and patients were deceived with some clinics for preventing and curing several diseases. In addition to the yield of exosomes, many other properties are affected by choice of isolation methods. Purity and physicochemical properties of exosomes are the major factors affected by different isolation methods. Different methods such as ultrafiltration, size exclusion chromatography, aqueous two-phase system, and polymer-based precipitation can be used for mass production of exosomes. The reaching large-scale desired exosomes for clinical usage is a main challenge. Yield of exosomes is typically less than 1 µg exosomal protein from 1 mL of culture medium [66, 67], while the beneficial dose of exosomes is about 10–100 µg exosomal protein/mouse in most researches [68, 69]. Use of exosomes in clinical trials necessity to conform to GMP[70]. The significant matters are prevailing in GMP for exosomes. First, large-scale production. One of the major problems for realizing exosome-based therapeutics is low productivity of exosomes. Second, collection of high-quality and uniform exosomes. Physicochemical and purity properties of exosomes are the major issues affected by diverse isolation methods. Third, standardization of storage conditions. Fourth, development of therapeutic potential of exosomes. For overwhelming low efficiency of exosomes, enlightening their therapeutic potential can be one possible elucidation. Overexpression or enrichment of therapeutic biomolecules is a simple way to progress therapeutic potential [71] (Fig. 5). In addition, delivery of exosomes into site of target tissue/cell is a critical issue. Since the biological effect of exosomes is employed by their uptake by target cells, clarification and control of biodistribution of exosomes are obligatory for the clinically therapeutic application. Five categories of cells such as bone marrow-derived MSCs, human cardiac progenitor cells, monocyte-derived DCs, adipose tissue-derived stem cells, and HEK293 cells have been used in GMP for the production of exosome [30]. There is less evidence on GMP manufacture for plant-derived exosomes, although several experimental studies have been documented [72, 73]. Overall, a normal clinical trial project and supervisory monitoring are noteworthy to confirm patient safety upon stem cells and exosomes treatment.

**Box 4:** Key questions about exosomes-based therapiesr

Which cell sources are promising for the treatment of diseases? For example, whether MSCs-derived exosomes are useful for most diseases or not. In addition, exosomes may induce adverse outcomes and trigger malfunction, therefore, which source of MSCs are safe for exosomes purification? Do other exosomes such as those of plant cells and bacteria have a superior advantage over animal ones? Do pre-conditioning or modifying exosome-producing cells adversely affect the efficacy of exosomes? Because these modifications may induce unwanted alterations in exosomes, the effects and safety of modified exosomes should be assessed independently What is the efficient isolation method to increase yield of exosomes? Different methods such as ultrafiltration, size exclusion chromatography, aqueous two-phase system, and polymer-based precipitation can be used for mass production of exosomes. Though, there is little information about difference in efficiency of exosome isolation among these methods What is a convenient and standard method for delivering exosomes to the damaged site? As known, exosomes may be clearance by macrophages located on the spleen or the lung tissues, which lower the efficacy of treatment What is a gold-standard method for purification exosomes? What is the best storage condition for saving exosomes? For therapeutic application, storage at higher temperatures is desirable because it does not require special equipment. Lyophilization of exosomes may expand their stability at higher temperatures Do exosomes incorporated into biomaterials have more advantages compared with intact exosomes?	

## Exosomes-based vaccine

Exosomes from tumor cells have been used for therapeutics to prompt strong anti-tumor immune responses. Furthermore, exosome-based vaccines showed hopeful outcomes against several kinds of infectious diseases. Exosomes from innate immune cells and tumor cells have the potential to be used as a vaccine for cancer [74] (Fig. 2). Several active molecules on exosomes like MHC and costimulatory molecules facilitate anti-tumor responses of immune cells. Recent progress in profiling exosomes cargo has also resulted in the increase of progressively active agents that may be possibly applied in cancer immunotherapies [75]. Based on a wide investigation into the function of exosomes in cancer immunotherapy, many pre-clinical studies have been performed with exosomes [75, 76]. In glioblastoma patients who receive anti-survivin immunotherapy, the low levels of CD9 + /SVN + and CD9 + /GFAP + /SVN + exosomes have been correlated with the sustained patient's survival[77]. Besides, tumor cell-derived exosomes contain DNA strands, which can induce immune cell responses by STING/cGAS pathway, and consequently, these exosomes may regulate tumor immunity with a possible role in checkpoint immunotherapy [78]. DCs-derived exosomes from cancer patients have been revealed safe and practicable for immunotherapy in some small clinical trials. DCs-exosomes immunotherapy results in a more accurate and precise immunity against cancer cells than other therapies. In addition, DCs-exosomes immunotherapy has more biostability and bioavailability, and lower costs [79]. In the first phase I clinical trial (NCT01159288), the authors performed a vaccination against metastatic melanoma using autologous DCs-derived exosomes administration and showed their safety of them. However, they did not observe profound CD4 + or CD8 + T cell responses; and it is still essential to examine the mechanism behind the vaccine antigen distribution [80]. Furthermore, in another study, DCs-derived exosomes have been shown to increase NK lysis activity and induce MAGE specific T cell responses in patients with NSCLC [81]. In an immunotherapy phase II clinical trial, authors aimed to boost T cell and NK immune responses in the progressive NSCLC patients by administration of IFNγ DCs-exosomes [82]. Authors reported that these exosomes could boost the antitumor immunity of NK cells in patients. However, the key clinical result, a progression-free survival (PFS) of 50%, could not be achieved and no outstanding immune responses were described in the study [82]. A last believable choice is to combine the DCs-exosomes vaccine with NK-based therapies [83], for producing synergistic immunogenic outcomes against NK-dependent tumors. Additionally, it seems that the construction of immortalized DC line library, which is modified to express a single MHC II molecule and/or no MHC proteins, may make constant production of DCs-exosomes promising, lessening therapy costs and the intervals of cell culture times. In brain cancer, which is resistant to immune cell recruitment, DCs-exosomes have been reported to be promising against glioma in mice model, proposing that these exosomes immunotherapy can be a novel therapy for glioblastoma [84]. Exosomes, based on the current investigational results and clinical trials, are thought to become immunotherapeutic vaccines for various cancers. Both clinical trials administered autologous DCs-exosomes that met their own current GMP standards. Such clinical trials to date are important for their demonstration of both the feasibility and the short-term safety of autologous exosomes administration, but safety concerns for therapies based on exogenous exosome-based products will debatably be more rigorous. Though some investigations have described protocols for bulk exosomes production and developments in biocompatibility [85, 86], additional preclinical and clinical scrutinize are necessary for confirmation. Finally, the comparatively smaller size and unified shape allow exosomes successfully escape clearance by the mononuclear phagocyte system, not only prolonging their circulation time but also implying their superiority in cell–cell communication.

**Box 5:** Key questions about exosomes-based therapies

What is large-scale constant preparation methods for DCs-derived exosomes? What are the specific international guidelines for the administration of the generation and application of immunotherapy? What is/are the pharmacokinetic of the DCs-exosomes in patients? Though these exosomes may influence T cell zones of secondary lymphoid organs successfully, they may have reached to DCs of the lymphatic sinus the macrophages of the subcapsular sinus or where they interact with innate lymphocytes. Therefore, the biodistribution of the administered DCs-exosomes requisite further exploration How to produce immortalized DCs line library for continuous producing exosomes? What is/are the best way/s to increase the efficiency of DCs-exosome in cancer therapy? Such combinational therapies as modified exosomes may increase the efficiency of cancer therapy	

## Conclusion

The EVs/exosomes field has made marvelous dives advancing in the past two decades. Many researchers are performing on exosomes discovery and application in human health offers. However, guidelines, standardization, nomenclature, and kinetics remain to progress into a more consistent approach. Facts of exosomes transfer in vivo and the mechanisms of cargo convey through body fluids and delivery into targets lag and are the field that we recommend necessitate further consideration. We principally inspire the collaboration of researchers from various disciplines such as clinicians, cell biologists, technology authorities, and computer experts can result in significant jumps in any assumed field. In this study, we carefully reviewed the therapeutic application and recent standing of clinical trials of exosomes according to potential application and disease type. Though preclinical studies have described hopeful outcomes, numerous improvements in exosome-based therapy are essential to acquire greater outcomes. Human and derived plant exosomes have been recorded in clinical trials. Exosomes from human cells comprise the majority and complete reports compared with those of plants. Clinical applications of exosomes comprise use as a biomarker for prognosis and diagnosing diseases, drug delivery carriers, cell-free therapy, and cancer vaccine. All of these applications are associated with challenges and limitations and need more exploration and standardization. Furthermost studies show human exosomes, conforming to GMP, to satisfy the use necessities of clinical trials. We think that exosomes efficacy in disease management mainly depends on the technological progress that allows their discovery, sensitivity, and specificity to distinct disease development.

## Data Availability

Data and materials are available upon request to the corresponding author.

## Abbreviations

EVs: Extracellular vesicles
MVs: Microvesicles
GMP: Good manufacturing practice
Abs: Apoptotic bodies
ECM: Extracellular matrix
CSF: Cerebrospinal fluid
BALF: Bronchoalveolar lavage fluid
PFS: Progression-free survival
ESCRT: Endosomal sorting complex required for transport
ILVs: Intraluminal vesicles
ISEV: International society for extracellular vesicles
MeeshoSCs: Mesenchymal stem cells
MVBs: Multivesicular bodies
NSCLC: Non-small cell lung cancer
SNAREs: Soluble NSF attachment protein receptors
